# Bacterial Outer Membrane Vesicles Loaded with Perhexiline Suppress Tumor Development by Regulating Tumor-Associated Macrophages Repolarization in a Synergistic Way

**DOI:** 10.3390/ijms241311222

**Published:** 2023-07-07

**Authors:** Shoujin Jiang, Wei Fu, Sijia Wang, Guanshu Zhu, Jufang Wang, Yi Ma

**Affiliations:** 1 School of Biology and Biological Engineering, South China University of Technology, Guangzhou 510006, China; 2 Guangdong Provincial Key Laboratory of Fermentation and Enzyme Engineering, South China University of Technology, Guangzhou 510006, China

**Keywords:** TAMs, M2, M1, repolarization, OMVs, perhexiline, immunomodulatory

## Abstract

Tumor-associated macrophages (TAMs) promote tumor development and metastasis and are categorized into M1-like macrophages, suppressing tumor cells, and M2-like macrophages. M2-like macrophages, occupying a major role in TAMs, can be repolarized into anti-tumoral phenotypes. In this study, outer membrane vesicles (OMVs) secreted by *Escherichia coli* Nissle 1917 carry perhexiline (OMV@Perhx) to explore the influence of OMVs and perhexiline on TAM repolarization. OMV@Perhx was internalized by macrophages and regulated the phenotype of TAMs from M2-like to M1-like efficiently to increase the level of tumor suppressor accordingly. Re-polarized macrophages promoted apoptosis and inhibited the mobility of tumor, cells including invasion and migration. The results indicate that OMVs improve the efficacy of perhexiline and also represent a promising natural immunomodulator. Combining OMVs with perhexiline treatments shows powerfully synergistic anti-tumor effects through co-culturing with re-polarized macrophages. This work is promising to exploit the extensive applications of OMVs and chemical drugs, therefore developing a meaningful drug carrier and immunomodulator as well as expanding the purposes of traditional chemical drugs.

## 1. Introduction

Cancer therapy remains a challenge, even though treatment methods have been developed to a large extent with the development of theory and technology. It is well known that tumorigenesis is a complex and dynamic process involving gene mutation, CSCs (cancer stem cells) renewal and differentiation, and cell–extracellular matrix interaction [1,2,3]. Recent research has defined tumor cells and the related extracellular environment as the tumor microenvironment (TME). Further research has shown that the TME primarily comprises tumor cells, stromal cells, the extracellular matrix (ECM), cytokines, and immune cells. According to the exit reports, the TME is heterogeneous and associated with chemoresistance, tumor metastasis, and prognosis [1,4]. Consequently, target therapy and regulating the status of the TME are widely appreciated among clinical specialists and scientists [5]. In the TME, tumor-associated immune cells play a pivotal role in tumorigenesis, metastasis, and treatment. Generally, immune cells in the TME consist of lymphocytes, dendritic cells, natural killer cells, neutrophils, myeloid-derived suppressor cells, and tumor-associated macrophages (TAMs) [5]. TAMs are categorized into two typical phenotypes comprised of classical-activated macrophages (M1) activated by lipopolysaccharide (LPS) and alternative-activated macrophages (M2) induced by interleukin 4 (IL-4) and take a principal part in TAMs [6]. Accordingly, M1 macrophages and secreting pro-inflammatory cytokines (IL-6, IL-12, TNF-α), are capable of promoting inflammation and inhibiting cancer development, whereas M2 macrophages that overexpress receptors (Arg-1, CD206) and anti-inflammatory cytokines (IL-4, IL-10, IL-13) have an anti-inflammatory and pro-tumorigenesis effect [7]. M1-related enzymatic makers include inducible nitric oxide synthase (iNOS), while receptor arginine-1 (Arg-1) is related to M2 [8]. M1 macrophages depend on glycolysis, fatty acid synthesis (FAS), and amino acid metabolism. Conversely, M2 macrophages utilize the TCA cycle and fatty acid oxidation (FAO) for a living [9]. Many studies have attempted to improve the efficacy of cancer therapies and inhibit tumor development by switching phenotypes of TAMs from M2 to M1 [10].

Due to shortcomings of chemical drugs, more and more non-tumor medicines have been explored to develop extensive applications, such as Metformin, Celebrex, Aspirin, and Disulfiram [11,12,13,14]. Some metabolic medicines have also been researched to modulate the phenotypes of TAMs. Oyarce et al. explored the influence of Perhexiline on TAM repolarization [15]. Perhexiline with hydrophobicity regulates repolarization with the help of IFN-γ by targeting and suppressing FAO in macrophages [16]. Cancer therapy based on nanotechnology is a burgeoning strategy thanks to the properties of nanoparticles, including their enhanced permeability and retention effect (EPR) and excellent drug-loading capability [17,18]. Meanwhile, biological nanoparticles and Gram-negative bacterial outer membrane vesicles (OMVs) with natural components and a size of 50–250 nm can activate the host immune system and load drugs [19]. Moreover, vaccines based on OMVs have been approved by the European Medicines Agency (EMA) and the Food and Drug Administration (FDA) [20]. OMVs can carry many drugs, especially hydrophobic drugs, which rely on electric charges and the ability to be retained on or cross through the membranes [21]. It is reported that OMVs can load paclitaxel, tegafur, and siRNA [10,22,23]. OMVs can also be modified by synthetic biological or chemical methods. OMVs presenting programmed death 1 (PD-1) can improve cancer immunotherapy via immune activation and checkpoint inhibition [24]. Shuang Qing et al. created a biomineralized OMV with an active-targeting ligand to improve antitumor therapy efficacy by targeting the TME in solid tumors [25].

OMVs as a platform have numerous advantages. However, we are unaware of the influence of OMVs on TAM polarization in vitro and whether traditional drugs have an antitumor effect with the help of OMVs or even exhibit a synergistic effect. Herein, we design perhexiline-loaded OMVs (OMV@Perhx) that carry perhexiline to TAMs, thereby regulating the substates of macrophages by activating M1 macrophages. Furthermore, repolarized macrophages execute the antitumor effect.

## 2. Results

### 2.1. Production and Characterization of Bacterial OMVs

**Figure 1:**
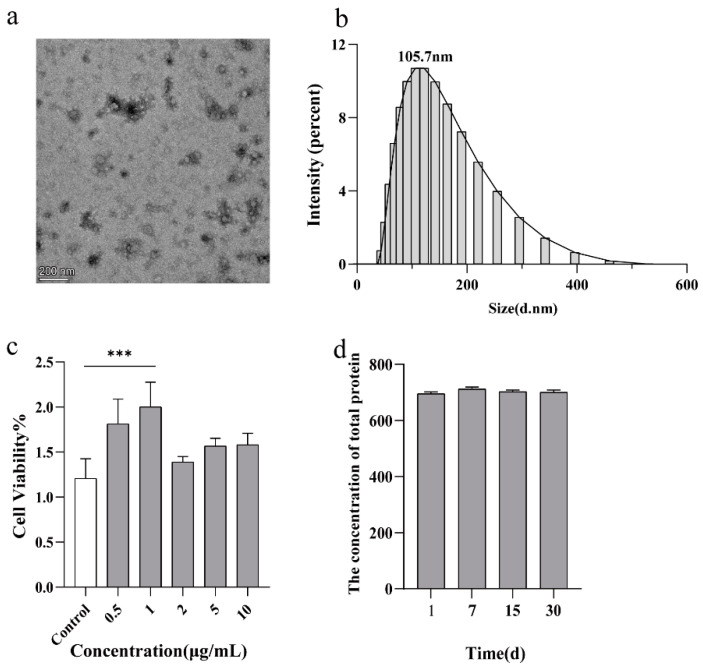
Characterization of OMVs. (**a**) Transmission electron micrograph image of *E*. *coli* Nissle 1917-derived OMVs. Scale bar, 200 nm. (**b**) Size distribution of OMVs was measured by dynamic light scattering analysis (n = 6). (**c**) Cell toxicity of OMVs was examined by Cell-Counting-Kit assay (n = 4). (**d**) The concentration changes of total protein were monitored by the BCA kit at different times (n = 6). Data were presented as the mean ± SD (one-way ANOVA comparison tests, *** *p* < 0.001).

Considering the toxicity of OMVs, we selected the commensal microorganisms probiotic *Escherichia coli* Nissle 1917, which is the main component of Mutaflor [26]. After successfully generating OMVs, TEM and DLS were used to characterize OMVs. TEM revealed that OMVs adopted a uniform spherical shape, and the average diameter of OMVs was 60.76 nm. (Figure 1a). In the meantime, DLS also showed the average diameter of OMVs was 91.28 nm (Figure 1b). In the meantime, we examined changes in the total protein concentration while OMVs moved from −20 °C to 4 °C; there were no significant differences from day 1 to day 30 (Figure 1d), indicating the stability of OMVs.

We then evaluated the safety of OMVs. The results are shown in Figure 1c and indicate that all concentrations we set were safe. In addition, low-concentration OMVs even promoted cell proliferation to a certain extent. We hypothesized that OMVs could be used as the metabolic substrate to regulate cell metabolism. Perhexiline showed no significant toxicity at low concentrations such as 2.5 μM and 5.0 μM (Figure S1). As a result, we selected OMVs at 2 μg/mL and perhexiline at 2.5 μM for further experiments.

### 2.2. Internalization of Drug-Loaded OMVs by Macrophages

**Figure 2:**
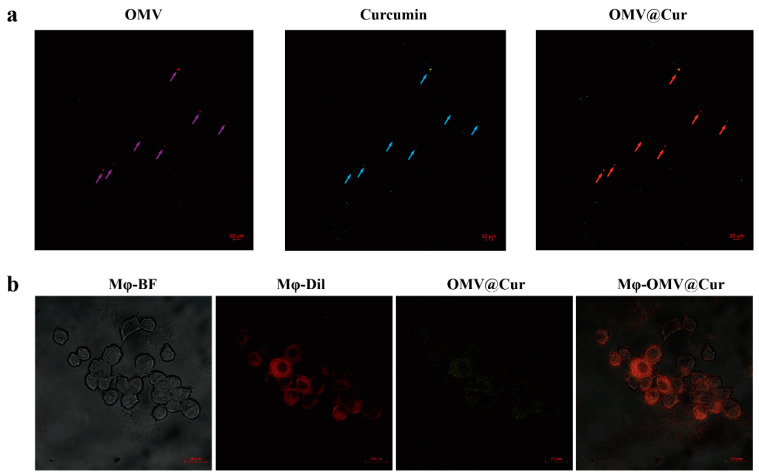
Curcumin-loaded OMVs and the internalization of OMV@Curcumin characterized by a confocal laser scanning microscope. (**a**) OMVs loading curcumin as imaged by CLSM; purple arrows, blue arrows, and red arrows represent Dil-labeled OMVs, curcumin, and OMV@Cur, respectively. Scale bar, 10 μm. (**b**) OMV@Cur internalized by macrophages. Red: Dil-stained macrophages; yellow: curcumin-labeled OMVs. Scale bar, 20 μm.

To visualize the successful combination of colorless perhexiline and OMVs, referring to a previous report [27], we selected curcumin (MW: 368.38) with yellow fluorescence instead of perhexiline (MW: 393.56). As has been reported, the conjunction of drugs and OMVs relies on properties such as hydrophobicity and molecular charge [21]. As shown in Figure 2a, OMVs and curcumin showed dense yellow and intense red fluorescence, respectively. As expected, OMV@Cur showed relevant orange fluorescence, and the Pearson correlation coefficient (PCC) was 0.768 (Figure S2), which indirectly indicated that OMVs could conjugate perhexiline successfully.

To confirm macrophages could internalize OMV@Cur, after securing the successful combination of curcumin and OMVs, the Dil work solution was changed to stain macrophages. Figure 2b showed that macrophages were stained with Dil. Meanwhile, OMVs combined with curcumin showed dense yellow fluorescence. Ultimately, macrophages presented noticeable orange fluorescence after merging; the PCCs were 0.665 (Figure S3), which implied that OMV@perhx could also be internalized efficiently by macrophages.

### 2.3. Macrophage Repolarization

**Figure 3:**
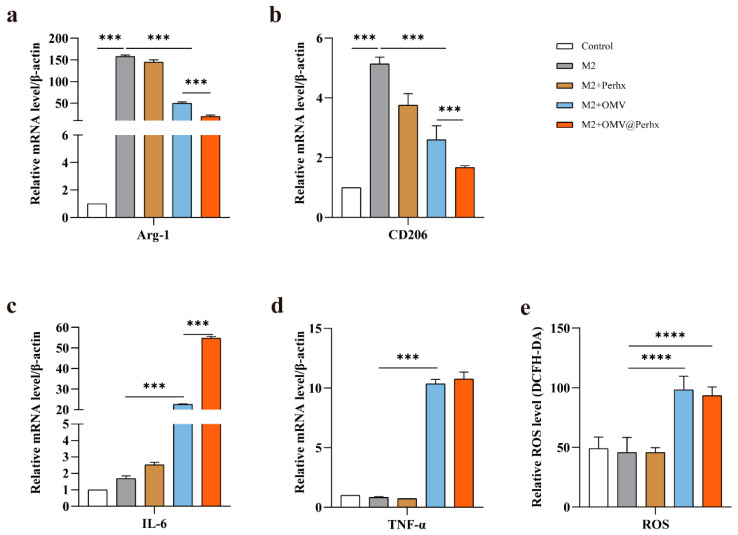
The level of polarization or repolarization was analyzed by qRT-PCR and ROS detection. (**a**,**b**) Transcription level of Arg-1 and CD206 was examined by qRT-PCR (n = 6). (**c**,**d**) IL-6 and TNF-α, biomarkers of M1-like polarization, were analyzed by qRT-PCR (n = 6). (**e**) Detection of relative ROS level in macrophages (n = 6). Control: PBS, M2: IL-4 (100 ng/mL), M2 + Perhx: IL-4 + perhexiline (2.5 μM), M2 + OMV:IL-4 + OMV (2 μg/mL), M2 + OMV@Perhx: IL-4 + OMV@Perhx (2 μg@2.5 μM). Data are presented as the mean ± SD (one-way ANOVA comparison tests, *** *p* < 0.001, **** *p* < 0.0001).

M2-like macrophages were detrimental to tumor progression, metastasis, and prognosis [28]. Here, we chose IL-6, TNF-α, and ROS as the biomarkers of M1-like macrophages, while Arg-1 and CD206 were selected as the M2-like biomarkers. The sequence of suitable primers is shown in Table S1. Firstly, we induced M2-like macrophages as a positive control upon IL-4 stimulation. As shown in Figure 3a,b, IL-4 increased M2-associated biomarkers, proving that M2-polarization was induced successfully. Next, OMVs or perhexiline was added into M2 macrophages for another 24 h treatment to repolarize macrophages.

As Figure 3a,b showed, compared with M2 macrophages, M2 macrophages treated with OMVs down-regulated transcriptional expression of M2-associated genes (Arg-1, CD206) significantly, especially for Arg-1. On the other hand, polarized-M2 macrophages treated with perhexiline had no significant changes in either Arg-1 or CD206. Nevertheless, M2 macrophages treated with OMV@Perhx inhibited M2-associated gene transcription in a more pronounced way than OMV-treated M2 macrophages, which implied a synergism between OMVs and perhexiline (Figure 3a,b). M1 biomarkers were also monitored simultaneously, Figure 3c,d shows OMVs or OMV@PerhX stimulation significantly increased transcriptional levels of IL-6 and TNF-α. Of note, the level of IL-6 was different between OMV and OMV@Perhx treatments. Combined with the first result, this shows a marked synergistic effect in co-applying OMVs and perhexiline. In the meantime, we verified the changes in ROS in macrophages, another typical marker of M1-like macrophages [29]. As shown in Figure 3e, IL-4-treated macrophages (M2) showed no significant differences compared with the control group, nor did the M2 + Perhx group. However, when OMVs or OMV@Perhx stimulated the M2-like macrophages, the level of ROS increased significantly (Figure 3e), which proved our hypothesis that OMV-related treatment could switch M2-like macrophages to M1-like macrophages.

**Figure 4:**
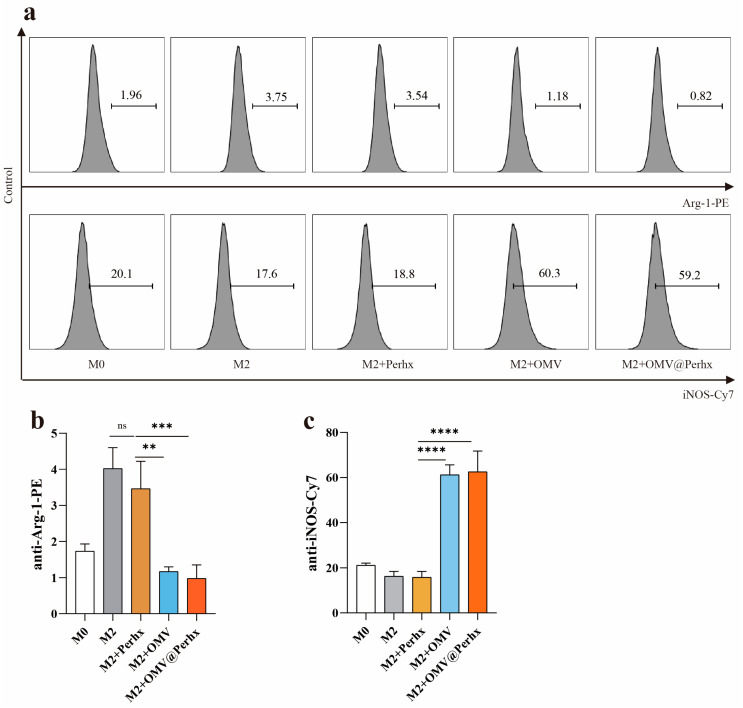
The polarization and repolarization were analyzed by flow cytometry. (**a**) iNOS-Cy7 and Arg-1-PE were biomarkers for M1 and M2, respectively; the level of biomarkers was quantified by flow cytometry. (**b**,**c**) Expression of biomarkers using histogram. Data are presented as the mean ± SD (one-way ANOVA comparison tests, ** *p* < 0.01, *** *p* < 0.001, **** *p* < 0.0001, n = 6).

To further verify the above results, a flow cytometry assay was conducted. As shown in Figure 4, the polarization and repolarization trends were consistent with the above results.

M2-associated biomarkers were down-regulated upon OMV or OMV@Perhx stimulation, and M1-associated biomarkers were promoted simultaneously, indicating TAMs were effectively regulated from M2 substates to M1 substrates. The results show that OMVs could reprogram TAMs from M2 to M1 effectively. In addition, perhexiline might be activated to result in immunomodulatory effects. Thus, there may be a synergism between OMVs and perhexiline.

### 2.4. Detection of Cytokines for Tumor Inhibition

Over the past 40 years, cytokines have been extensively studied as either cancer targets or treatments [30]. Among numerous cytokines and chemokines, IFN-γ has shown to be vital for immunity, potentiating NO and CXCL10 secretion to reinforce antitumor effects. In the meantime, NO secreted by macrophages can kill tumor cells directly and improve the impact of TLR agonists to inhibit tumor progress [31,32].

**Figure 5:**
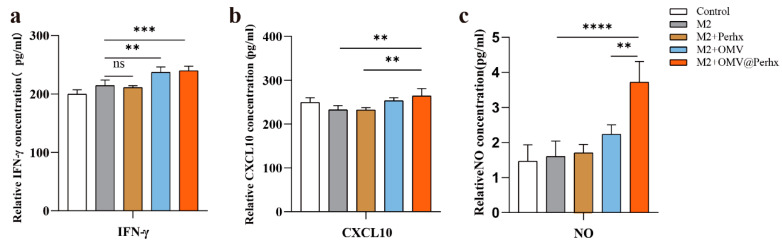
The level of tumor suppressors secreted by diverse phenotypes of macrophages. (**a**) The relative IFN-γ concentration in cell-cultured supernatant analyzed by an ELISA kit (n = 6). (**b**) The relative CXCL10 concentration in cell-cultured supernatant analyzed by an ELISA kit (n = 6). (**c**) The level of NO in cell-cultured supernatant analyzed by Griess NO detection kit (n = 6). Control: PBS, M2: IL-4 (100 ng/mL); M2 + Perhx: IL-4 + perhexiline (2.5 μM); M2 + OMV: IL-4 + OMV (2 μg/mL); M2 + OMV@Perhx: IL-4 + OMV@Perhx (2ug@2.5 μM). Data are presented as the mean ± SD (one-way ANOVA comparison tests, ** *p* < 0.01, *** *p* < 0.001, **** *p* < 0.0001).

As a result, we measured the amounts of IFN-γ, CXCL10, and NO secreted by polarized and repolarized M1-like macrophages. As shown in Figure 5a, a single treatment with perhexiline resulted in no apparent changes in IFN-γ. While treatment with OMVs and OMV@Perhx resulted in IFN-γ increasing significantly, which was consistent with our hypothesis. A similar trend was observed in CXCL10 (Figure 5b), and the level of CXCL10 treated with OMV@Perhx was higher than that treated with Perhx. In addition, the level of NO was also enhanced significantly upon OMV or OMV@Perhx stimulation as compared with the positive group or perhexiline-treated group (Figure 5c). It was also expected that M2-like macrophages treated with OMV@Perhx showed a synergism compared with OMV treatment (*p* < 0.01) and Perhx treatment (*p* < 0.01).

### 2.5. Tumor Apoptosis Assay

**Figure 6:**
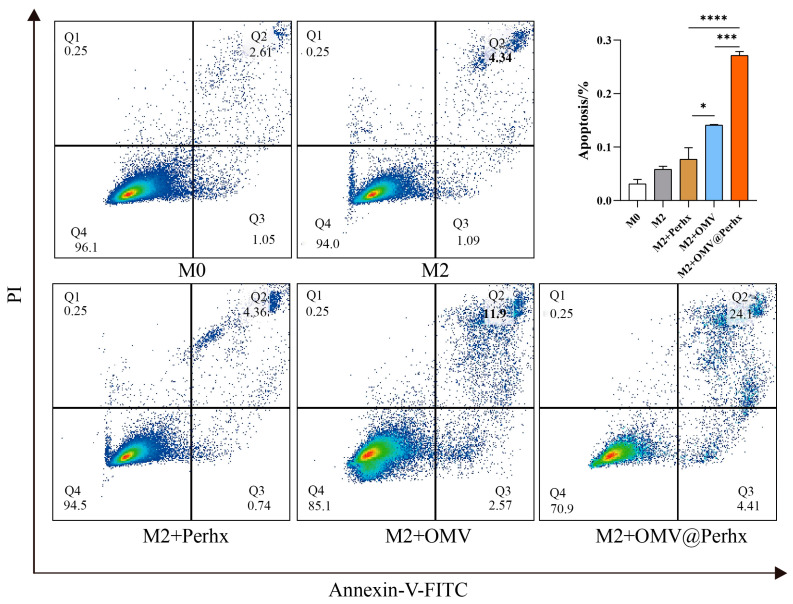
The apoptosis level of CT26 cells treated with different macrophage-cultured supernatant. M0: macrophages + PBS (24 h); M2: macrophages + IL-4 (24 h); Perhx: macrophages + IL-4 (24 h) + Perhx (24 h); OMV: macrophages + IL-4 (24 h) + OMV (24 h); OMV@Perhx: macrophages + IL-4 (24 h) + OMV@Perhx (24 h). Data are presented as the mean ± SD (one-way ANOVA comparison tests, * *p* < 0.05, *** *p* < 0.001, **** *p* < 0.0001, n = 6).

According to designs, the repolarized macrophages can suppress tumor development. Sequentially, as shown in Figure 6, the apoptosis of CT26 showed a pronounced upward tendency after OMV-related stimulation. Among all the experimental groups, perhexiline-treated macrophages had negligible influence on CT26 tumor apoptosis. However, OMVs and OMV@Perhx stimulation could significantly induce tumor apoptosis, and the influence of OMV@Perhx stimulation was stronger than OMV stimulation. At this time, the synergistic effect of co-application was remarkable, even if the proapoptotic impact of OMVs was also significant.

### 2.6. Tumor Migration and Invasion Assay

To further authenticate that the repolarized macrophages could inhibit tumor development, the transwell co-cultured system was adopted to analyze the migration and invasion of CT26. Tumor cells caused secondary cancer by invading and migrating to other organs where M2 macrophages secreted matrix metalloproteinases (MMP), serine proteases (SP), and cathepsins (CE) to modify cell–cell junctions and disrupt the basal membrane [33]. In the transwell system, both the transwell migration and invasion assay used an 8 μm polyester membrane. The only difference was that the transwell invasion assay covered the membrane with Matrigel to simulate the basal membrane in vivo.

**Figure 7:**
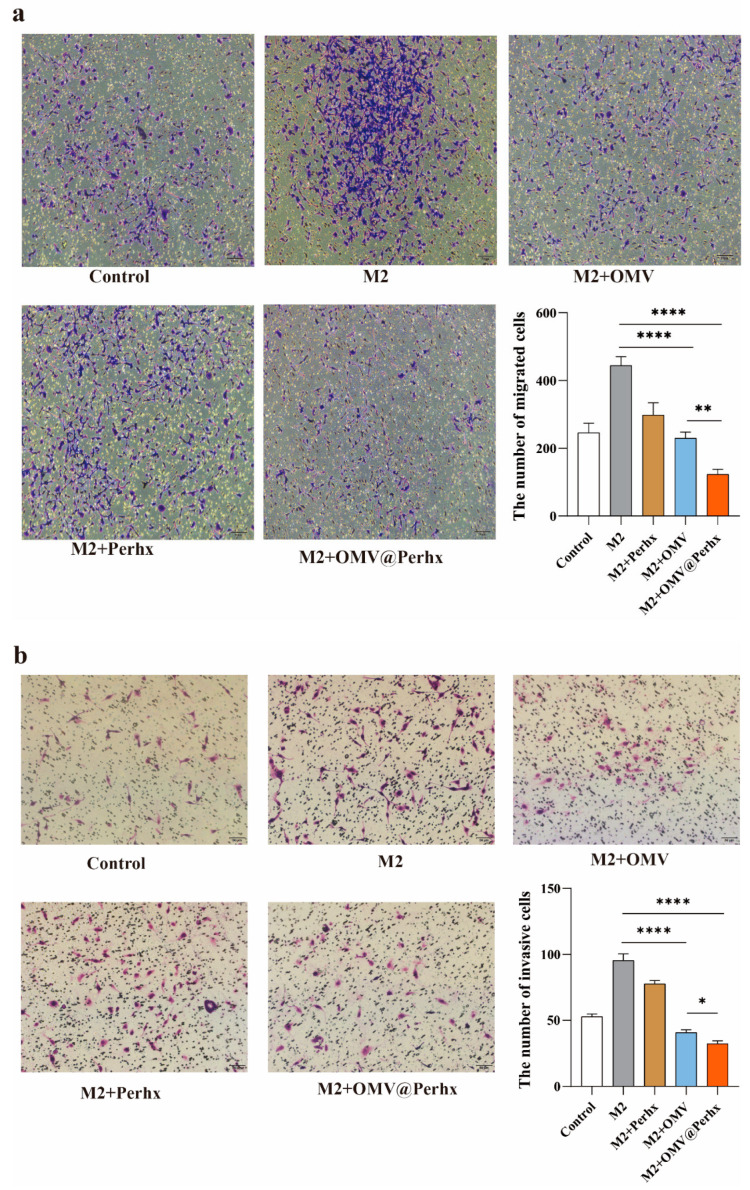
The influence of repolarized macrophages on the mobility of CT26 tumor cells as verified by transwell assay. (**a**) Transwell migration assay. Macrophages stimulated with different treatments, including PBS, IL-4, IL-4 + OMVs, IL-4 + perhexiline, and IL-4 + OMV@Perhx, show distinct effects on the migration ability of CT26 tumor cells (n = 6). (**b**) Capacity of CT26 tumor cells to cross through the Matrigel as examined by transwell invasion assay upon different treatments (PBS, IL-4, IL-4 + OMVs, IL-4 + perhexiline, IL-4 + OMV@Perhx, n = 6). Data are presented as the mean ± SD (one-way ANOVA comparison tests, * *p* < 0.05, ** *p* < 0.01, **** *p* < 0.0001). Scale bar = 50 µm.

In the transwell migration assay (Figure 7a), IL-4-treated macrophages performed an enhanced invasive capacity for CT26 tumor cells, indicating that M2 macrophages promoted tumor mobility. In contrast with the M2-like positive group, OMV-treated and OMV@Perhx-treated M2 macrophages reduced the invasive ability of CT26. Simultaneously, a similar result was shown in the transwell invasion assay (Figure 7b). When co-cultured with OMV- or OMV@Perhx-treated M2-like macrophages, invaded tumor cells declined remarkably. It was suggested that OMV or OMV@Perhx treatment could switch macrophage phenotypes from M2 to M1, thereby executing the antitumor effect. Moreover, compared with OMV-treated groups, the regulatory capacity of OMV@Perhx was superior in the migration and invasion assay.

## 3. Discussion

In the field of tumor therapy, numerous therapeutic theories and methods have emerged, providing insights into tumor progression and treatment. For instance, traditional opinions theorized that tumor cells originated from mutated body cells; however, the latest views are that cancer might come from CSCs [34]. Besides these two theories, there are also other views related to tumor initiation [35]. Likewise, it is widely accepted that changing the TME is crucial to cancer immunotherapies. In our research, TAM modulation and nanomaterial therapy are significant due to the plasticity of TAMs and the outstanding properties of nanomaterials.

In this study, we selected a traditionally clinical hydrophobic drug (perhexiline: impairs fatty acid transport into mitochondria through CPT-1 inhibition [16]) instead of the prevailing metabolic-pathway-targeting drugs to exploit new options for altering the immunosuppressive microenvironment for cancer immunotherapy. Meanwhile, OMVs were selected to modulate TAM repolarization by loading and activating perhexiline. Based on our designs, OMVs were not only regarded as a carrier of perhexiline but also as an immune regulator of the TME and a promotor of perhexiline. It has been reported that when IFN-γ is absent, the metabolic drug perhexiline is ineffective [15]. We assumed that OMVs could promote the secretion of IFN-γ and other molecules, thereby activating perhexiline and inhibiting tumor growth. As the results showed, tumor suppression increased markedly upon OMV-related stimulation, while a single perhexiline treatment had a poor effect, both in regulating phenotypes and in tumor mobility. Whereas when perhexiline was partnered with OMVs, it caused many changes. Compared with a single treatment, OMV@Perhx treatment inhibited polarization and induced repolarization significantly; therefore, tumor mobility and viability were restrained remarkably. In addition, it was demonstrated that therapy had a synergistic effect on OMVs and perhexiline, which was in line with our expectations. Even though some M1-associated molecules increased insignificantly when compared with the OMV-treated group or Perhx-treated group, such as ROS and TNF-α, we assumed that this might be connected with the limited influence of perhexiline.

According to our findings, OMVs maintained safety and stability in vitro, which is indispensable for immunotherapies and drug loading [36]. We assumed that OMVs could act as an agonist of toll-like receptors (TLRs) to activate an immune response by skewing M2-like macrophages to M1-like macrophages, thereby regulating the immunosuppressive status of the TME. As is well known, most clinical patients show a poor effect on immune activation due to a lack of positive immune cells [37]. More and more researchers have attempted to alter “cold tumors” to “hot tumors” by changing the immunosuppressive state of immune cells, inclusive of M2-like macrophages. Although we confirmed the immunomodulatory and synergistic effects of OMVs and perhexiline, metabolic pathway investigations are lacking. Oyarce et al. explored the TCA and FAO metabolic changes in macrophage polarization using different drugs [15]. Jha et al. used transcriptome and metabolomic analyses to research the polarization network [38]. With deeper investigation, other pathways or molecules related to bacteria-based immunomodulation may be found, which has guiding significance for scientific research and clinical practice. In future studies, we can explore the pathways associated with OMVs and immunomodulation. Moreover, OMVs secreted by other bacteria are attractive, not just probiotics. Diverse bacteria have different habits and characteristics; for instance, gastrointestinal flora is closely related to cancer, and the effects of the outer membrane vesicles of gastrointestinal flora on cancer therapy and the gastrointestinal immunity system are meaningful and influential. It was reported that gastrointestinal flora could improve PD-1 treatment significantly [39]. By using other cancer-associated bacteria, including the tumor-resident intracellular microbiota [40], the synergism may be more powerful. Many bacteria have specific preferences that can be taken into consideration; e.g., Salmonella can target tumor surroundings because of its anaerobic nature [41].

## 4. Materials and Methods

### 4.1. Cell Culture

RAW264.7 murine macrophages and CT26 murine colon adenocarcinoma cells were grown in Dulbecco’s modified Eagle medium supplemented with 10% Fetal Bovine Serum (Procell, Wuhan, China) and 1% Antibiotic-Antimycotic (Gibco, New York, NY, USA). All cells were incubated at 37 °C in an atmosphere containing 5% CO_2_.

### 4.2. OMV Preparation and Characterization

OMVs were prepared following the improved protocol described previously. Briefly, *E. coli* Nissle 1917 was cultured in fresh lysogeny broth until OD600 reached 1.0 (37 °C, 220 rpm). The medium was centrifuged (10,000× *g*, 10 min, 4 °C), and the supernatant was passed through a 0.45 μm filter and concentrated using a 100 kDa ultrafiltration cube. The solution was pelleted by ultracentrifugation (170,000× *g*, 1.5 h, 4 °C). Consequently, the pellet was resuspended in PBS and filtered (0.22 μm) to ensure intact bacteria and cell debris were eradicated. Finally, the collected OMVs were stored at −20 °C. The total protein concentration of OMV preparations was quantified using the bicinchoninic acid assay (Thermo Scientific, Waltham, MA, USA). The morphology of OMVs was verified using transmission electron microscopy (TEM), and the size of OMVs was monitored by dynamic lighting scattering (DLS) analysis with a Zetasizer Nano S90 instrument.

### 4.3. Cell Viability Assay

The cell toxicity of OMVs was examined using the colorimetric cell counting kit-8 assay according to the manufacturer’s instructions.

### 4.4. Drug Loading and Internalization

To verify the drug was loaded on OMVs, OMVs (200 μg/mL) and curcumin (50 μM) were incubated for 30 min at 37 °C and mixed adequately. Curcumin was used to take the place of perhexiline as curcumin has similar properties (hydrophobicity, molecular weight) to perhexiline. Dil working solution (Beyotime, Shanghai, China) was used to stain OMVs. Afterward, the solution (OMV@Cur-Dil) was ultracentrifuged at 150,000× *g* for 1.5 h at 4 °C to remove the unloaded drug and dye. Finally, a confocal laser scanning microscope confirmed the combination and internalization of OMV@Cur (Zeiss LSM800).

### 4.5. Polarization and Repolarization

For M0 to M2/M1 polarization, RAW264.7 was seeded into 12-well flat-bottom plates at a density of 8 × 10^4^ cell/well. After a 12 h culture, cells were treated with IL-4 (100 ng/mL, PeproTech, Cranbury, NJ, USA) for 24 h. For M2 to M1 repolarization, M2 was treated with perhexiline (5 μΜ, Topscience, Shanghai, China), OMVs (2 μg/mL), and OMV@Perhexiline (2 μg/mL) for 24 h.

qRT-PCR, flow cytometry analysis, and ROS assay were used to identify the phenotype of macrophages. Arginine receptor (Arg-1, anti-Arg1-PE, Thermo Fisher, Waltham, MA, USA) and inducible nitric oxide synthase (iNOS, anti-iNOS-PE-Cy7, Thermo Fisher, MA, USA) were thought to be M2 and M1 biomarkers for flow cytometry analysis, respectively. As for qRT-PCR methods, Arg-1 and mannose receptor (CD206) were the classical genes of M2; and the expressions of IL-6, TNF-α, and ROS were biomarkers of M1. The RNA Extraction Kit and reverse transcriptase were purchased in Vazyme, Najin, China, and the ROS assay kit was purchased in Beyotime, Shanghai, China.

### 4.6. ELISA Assay and NO Assay

The expression of IFN-γ, CXCL-10, and NO was examined by commercial kits (LunChangShuoBiotech, Xiamen, China), and all steps followed the manufacturer’s instructions strictly.

### 4.7. Cell Apoptosis Assay

An Annexin-V-PI kit (Beyotime, Shanghai, China) was used to examine the cell apoptosis level. Product descriptions guided all steps, and all samples were analyzed by flow cytometry (BD Biosciences, Franklin Lakes, NJ, USA) within 1 h.

### 4.8. Transwell Assay

Before co-culturing, the steps of repolarization were the same as the above descriptions. Simultaneously, CT26 starved for 12 h was seeded into a transwell chamber at a density of 1 × 10^5^. After co-culturing for 24 h, the transwell chambers were washed three times with PBS. Then, CT26 was fixed with 4% paraformaldehyde for 20 min, except those cells wiped away with a cotton swab in the upper chamber. Then, 0.1% crystal violet was used to stain cells for 30 min. Following this, five fields in each section were selected randomly selected for photographing and counting under a microscope. Of note, the transwell invasion assay required covering the chamber with Matrigel diluted with a serum-free medium at a ratio of 1:8 in advance.

### 4.9. Statistical Analysis

Experimental data are presented as the mean ± SEM of the number of experiments, indicated as “n”. For the determination of significance, a one-way analysis of variance was used (ANOVA) followed by Fisher’s LSD post hoc using Prism 8 (GraphPad. San Diego, CA, USA). A *p*-value of 0.05 was considered a statistically significant difference between the compared data (* = *p* < 0.05, ** = *p* < 0.01, *** = *p* < 0.001, and **** = *p* < 0.0001).

## 5. Conclusions

In summary, we exploit a powerful platform for TAM repolarization and drug loading and activation. This study shows that *E. coli* Nissle 1917 can generate sphered OMVs, sized 60.76 nm on average. OMVs can transport perhexiline to macrophages and repolarize macrophages from M2 to M1 effectively as well as activate the regulatory effect of perhexiline on macrophage repolarization. More importantly, OMVs and perhexiline have a synergistic effect on inhibiting CT26 cell development by co-culturing with repolarized macrophages, inclusive of promoting apoptosis, inhibiting invasion and migration. According to our expectations, OMVs will attract more attention to TME regulation and cancer immunotherapy. On the other hand, additional clinical hydrophobic drugs can be exploited to modulate TAM repolarization, even for TME regulation, thereby exploring a broader market for ameliorating cancer therapy.

## Data Availability

The raw data supporting the conclusions of this article will be made available by the authors, without undue reservation.

## Supplementary Materials

The supporting information can be downloaded at: https://www.mdpi.com/article/10.3390/ijms241311222/s1.
