# Supplementary material for: Bacterial Outer Membrane Vesicles Loaded with Perhexiline Suppress Tumor Development by Regulating Tumor-Associated Macrophages Repolarization in a Synergistic Way
Source: Int J Mol Sci. 2023 Jul 7;24(13):11222. doi: 10.3390/ijms241311222 (PMC10342243; doi:10.3390/ijms241311222)
Supplement: Supplementary file 1 [file ijms-24-11222-s001.zip › ijms-2469938-supplementary.pdf]

Supplementary Materials

of

Bacterial Outer Membrane Vesicles Loaded with Perhexiline Suppress Tumor Development by Regulating Tumor-associated Macrophages Repolarization in a Synergistic Way

Shoujin Jiang[1], Wei Fu[1], Sijia Wang[1], Guanshu Zhu[1], Jufang Wang[1,2], Yi Ma[1,2*]

*** Correspondence:**

Corresponding Author: bimayikobe@scut.edu.cn

Supplementary Figures and Tables

Supplementary Table S1. Fluorescence quantitative PCR primer sequence

Primer	Sequence (5'-3')
β-actin	F： CACTGTCGAGTCGCGTCC
	R： CGCAGCGATATCGTCATCCA
Arg-1	F： ACATTGGCTTGCGAGACGTA
	R： ATCACCTTGCCAATCCCCAG
CD206	F：GTGGGGACCTGGCAAGTATC
	R： CACTGGGGTTCCATCACTCC
IL-6	F： CCCCAATTTCCAATGCTCTCC
	R： CGCACTAGGTTTGCCGAGTA
TNF-α	F： TAGCCCACGTCGTAGCAAAC
	R： ACAAGGTACAACCCATCGGC

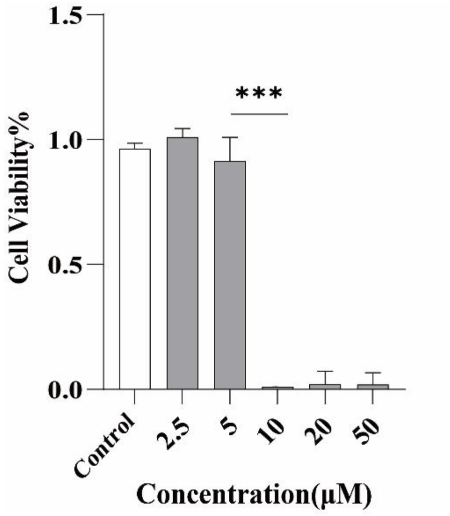

Supplementary Figure S1. The cytotoxicity of perhexiline to RAW264.7 was monitored by CCK-8 (n=6). Data were presented as the mean±SD (one-way ANOVA comparison tests, ****P*<0.001).

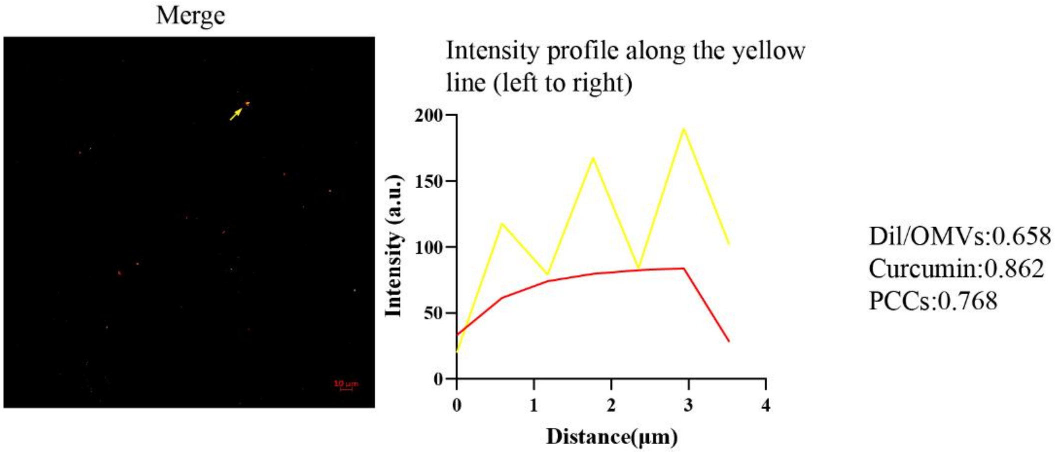

Supplementary Figure S2. Colocalization of Dil-Cur in OMVs. OMVs were treated with Dil and Curcumin, and imaged by CLSM. Pearson's correlation coefficients for Dil and Cur are 0.658 and 0.682, respectively. Excitation wavelength for Dil and Cur are 610 and 517 nm, respectively. Scale bar, 10 μm.

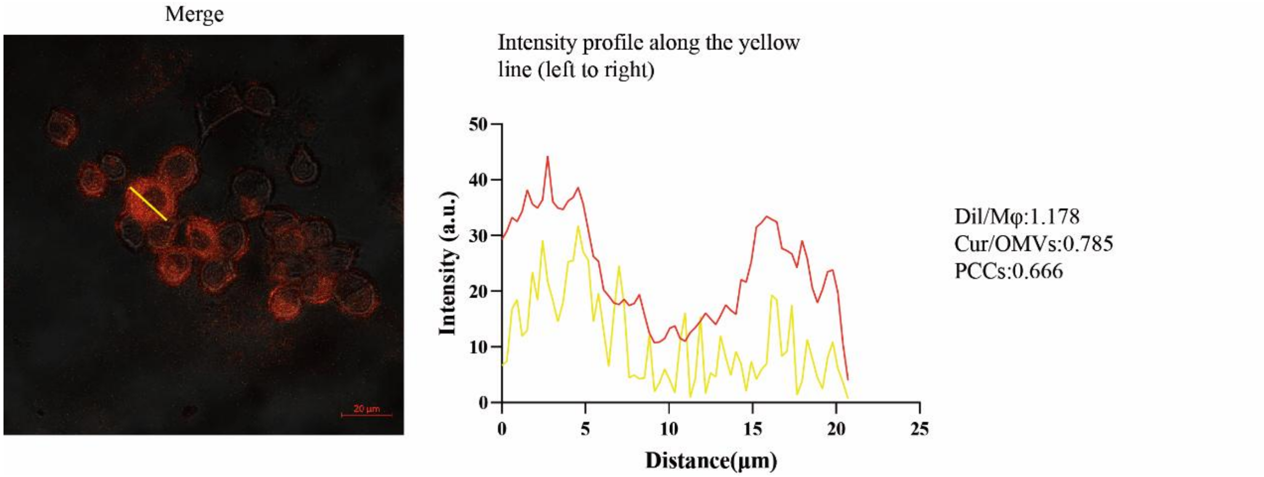

Supplementary Figure S3. Colocalization of Dil-Cur in macrophages. OMVs were treated with Curcumin, and macrophages were treated with Dil. Pearson's correlation coefficients for Dil and Cur are 1.178 and 0.785, respectively. Excitation wavelength for Dil and Cur are 610 and 517 nm, respectively. Scale bar, 20 μm.
